# Effects of the FNDC5/Irisin on Elderly Dementia and Cognitive Impairment

**DOI:** 10.3389/fnagi.2022.863901

**Published:** 2022-03-31

**Authors:** Jin Peng, Jinhui Wu

**Affiliations:** National Clinical Research Center for Geriatrics, Department of Gerontology and Geriatrics, West China Hospital, Sichuan University, Chengdu, China

**Keywords:** dementia, irisin, muscle brain cross talk, BDNF, inflammation, oxidative stress

## Abstract

Population aging is an inevitable problem nowadays, and the elderly are going through a lot of geriatric symptoms, especially cognitive impairment. Irisin, an exercise-stimulating cleaved product from transmembrane fibronectin type III domain-containing protein 5 (FNDC5), has been linked with favorable effects on many metabolic diseases. Recently, mounting studies also highlighted the neuroprotective effects of irisin on dementia. The current evidence remains uncertain, and few clinical trials have been undertaken to limit its clinical practice. Therefore, we provided an overview of current scientific knowledge focusing on the preventive mechanisms of irisin on senile cognitive decline and dementia, in terms of the possible connections between irisin and neurogenesis, neuroinflammation, oxidative stress, and dementia-related diseases. This study summarized the recent advances and ongoing studies, aiming to provide a better scope into the effectiveness of irisin on dementia progression, as well as a mediator of muscle brain cross talk to provide theoretical support for exercise therapy for patients with dementia. Whether irisin is a diagnostic or prognostic factor for dementia needs more researches.

## Introduction

The world has entered an aging society. In addition to chronic diseases, the elderly is accompanied by a series of geriatric symptoms. Cognitive impairment is a classic symptom of geriatric syndrome, which occurs from mild cognitive impairment (MCI) to dementia (Sanford, 2017). MCI is an intermediate state between neurotypical cognition and neurodegenerative dementia (Petersen et al., 2018). The prevalence of MCI in the elderly population aged ≥60 years is approximately 6.4–25% and increases with age according to the American Academy of Neurology (AAN) guidelines (Cheng et al., 2017). Dementia is the most serious form of cognitive impairment; diminishes the physical and mental function of older people, quality of life, and disability; and is the fifth leading cause of death (Winblad et al., 2004).

There were some risk factors of MCI and dementia, such as cardiovascular diseases (Schumacher et al., 2013), inflammation (Huh et al., 2014), and stroke (Chen et al., 2019). Alzheimer’s disease (AD) is a classic type of dementia, which is characterized at the neuropathological level by deposits of insoluble amyloid β-peptide (Aβ) in extracellular plaques and aggregated Tau proteins (Hodson, 2018). Developing evidence suggested that decreased brain-derived neurotrophic factor (BDNF) (Amidfar et al., 2020) and damaged synaptic plasticity (Skaper et al., 2017) led to dementia. However, the mechanism remains to be clarified.

Irisin, a myokine containing 112 amino acids, is secreted by skeletal muscle after exercise stimulation, which was first found in 2012 by Boström et al. (2012). It is processed from the type I membrane protein encoded by the FNDC5 gene, then secreted into the blood and circulated to several systems, and passed through the blood-brain barrier (BBB). Irisin consists of an N-terminal fibronectin III (FN III)-like domain attached to a flexible C-terminal tail and a continuous inter-subunit β-sheet dimer (Mahgoub et al., 2018). This structure is stabilized because of the hydrogen bonds and its interactions between the side chains of adjacent subunits, especially between Arg-75 and Glu-79, thus protecting the dimer ends and Trp-90/Trp-90 (Schumacher et al., 2013). Peroxisome proliferator-activated receptor γ (PPARγ) coactivator-1 α (PGC-1α) is the main regulator of FNDC5 in skeletal muscles in rodents and humans (Huh et al., 2014). Endurance exercise activates on PGC-1α to induce cleavage of FNDC5 to irisin. PGC-1α interacts with a wide range of transcription factors, and it is expressed in skeletal muscle, heart, and brain (Lin et al., 2002). It interacted with several pathways such as the p38 mitogen-activated protein kinase (MAPK) pathway stimulated by exercise (Akimoto et al., 2005), 5’ adenosine monophosphate-activated protein kinase (AMPK) pathway (Chen et al., 2019), Sirtuin1 (Sirt1) pathway (Safarpour et al., 2020), and the cyclic adenosine monophosphate (cAMP) response element-binding (CREB) pathway. The cAMP-mediated PGC-1α/CREB signaling bolstered the expression of FNDC5 (Yang et al., 2018). Besides, FNDC5 and irisin expressed in many tissues, such as skeletal muscle, pancreas, brown adipose tissue (BAT), liver, and brain, especially in the hippocampus and hypothalamus, are important for memory and cognition (Dun et al., 2013; Varela-Rodríguez et al., 2016).

Irisin was associated with various metabolic diseases such as diabetes, cardiovascular disease, and obesity (Polyzos et al., 2018). It induced the expression of mitochondrial uncoupling protein 1 (UCP1) (Castillo-Quan, 2012), increasing thermogenesis and converting white adipose tissue (WAT) into BAT. Furthermore, irisin exerted favorable effects on glucose metabolism to maintain glucose homeostasis and improve insulin resistance, of which mechanisms involved β cell regeneration (Natalicchio et al., 2017), reducing gluconeogenesis and promoting glycogen synthesis (Polyzos et al., 2013; Roca-Rivada et al., 2013). Besides, irisin performed a protective function on lipid metabolism involving several pathways such as the AMPK-SREBP2 pathway (Tang et al., 2016). It was also antioxidative, anti-inflammatory, and attenuating apoptosis, functioning to alleviate mitochondrial dysfunction (Mazur-Bialy et al., 2017b; Tu et al., 2020; Zhang et al., 2020). Many studies have reported that irisin had neuroprotective functions in AD (Kim and Song, 2018; Lourenco et al., 2019; de Freitas et al., 2020). Lourenco et al. (2019) elucidated that FNDC5/irisin was decreased in AD brains and CSF and in AD experimental models, but there was no significance in plasma irisin levels. Conti et al. (2019) reported a slight increase in irisin serum levels in patients with AD. Zhang et al. (2021) suggested that serum irisin might be a biomarker of cognitive decline in vascular dementia. Bičíková et al. (2021) reported that movement was a positive modulator of aging and the PPARγ is a critical link between mental function and aging. FNDC5/irisin is stimulated by PGC-1α, indicating irisin might be the mediator of muscle and brain cross talk. Some clinical observations and mechanisms were reported.

We try to summarize the research on the relationship between irisin and cognitive impairment and to understand the mechanisms of direct neuroprotective and indirect risk reduction. This study intended to explore whether irisin is a potential serum predictor of cognitive impairment in the elderly and an underlying mediator of muscle-brain cross talk to support exercise therapy for patients with dementia.

## FNDC5/Irisin in Muscle-Brain Cross Talk

Accumulating evidence is supporting the existence of muscle brain cross talk, a muscle-brain endocrine loop (Pedersen, 2019). Brain sensed exercise indirectly *via* adiponectin and liver-derived proteins such as fibroblast growth factor 21 (FGF21) and insulin-like growth factor 1 (IGF1), and muscle secreted myokines to regulate the brain function as a loop. The exercise was believed to decrease the risk of dementia (Santos-Lozano et al., 2016), delay the cognitive decline in patients with neurodegenerative disorders and prevent stress, anxiety, and depression (Pedersen and Saltin, 2015). The underlying mechanism might be the muscle brain cross talk. The physical activity enhanced circulating levels of myokines to enable the direct cross talk of muscle and brain, affecting neuronal proliferation and differentiation, synaptic plasticity, memory, and learning (Scisciola et al., 2021).

**FIGURE 1:**
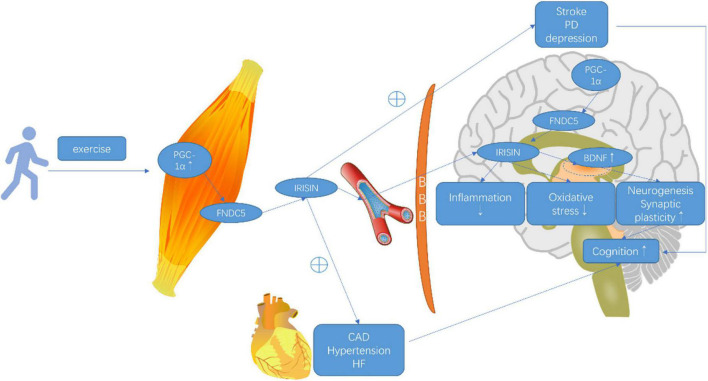
Effects of FNDC5/irisin on elderly dementia and cognition. Exercise promoted PGC-1α, which stimulated fibronectin type III domain-containing protein 5 (FNDC5) to be dissected into irisin. Irisin was shed into a blood vessel, circulating the whole body and passing through blood–brain barrier. Irisin might increase the expression of brain-derived neurotrophic factors, which improved synaptic plasticity, neuronal survival, neuronal differentiation, and neuronal health, thus cognition. FNDC5 was also highly expressed in hippocampus and stimulated by peroxisome proliferator-activated receptor γ (PPARγ) coactivator-1 α (PGC-1α) to be cleaved into irisin. Besides, irisin acts with its anti-inflammation and anti-oxidative effects to defend cognition deficits. Furthermore, FNDC5/irisin might have positive connections to dementia-related diseases, such as coronary artery disease, hypertension, heart failure, stroke, and Parkinson’s disease, and depression to protect against dementia.

The exercise was tightly related to the PGC1-α/FNDC5/BDNF pathway. FNDC5 gene expression was elevated following the increased PGC-1α expression induced by exercise both in central and peripheral organs, which stimulated the expression of BDNF in the brain (Boström et al., 2012). Irisin, as a myokine dissected from FNDC5, was also mediated by PGC-1α and passed through the BBB to increase the BDNF expression and enhance learning, memory, and mood (Lourenco et al., 2019). On the one hand, periphery irisin delivered to the brain and overexpressed irisin in the brain increased BDNF. On the other hand, knockdown of FNDC5 reduced the central BDNF expression (Severinsen and Pedersen, 2020). Figure 1 elucidated that irisin acted as a mediator of muscle brain cross talk and the effects of FNDC5/irisin on elderly cognition.

## FNDC5/Irisin Act on CNS

### FNDC5/Irisin and Neurogenesis

Brain-derived neurotrophic factor expresses highly in the brain, and it has considerable effects on synapses (Lu et al., 2014). It, mostly released from microglia and astrocytes, acts to promote synaptic plasticity, neuronal survival, neuronal differentiation, and neuronal health (Binder and Scharfman, 2004; Zuccato and Cattaneo, 2009). It was well-related to neurofunction and cognition. BDNF is bound to tropomyosin-related kinase B (TrKB) receptor to exert considerable effects. Decreased BDNF/TrkB activity resulted in neurodegeneration. Downregulation of BDNF/TrkB caused neuroinflammation, increasing inflammatory cytokines such as IL-1β and IL-6. Then triggered the JAK2/STAT3 pathway, resulting in the upregulation of C/EBPβ/AEP signaling, which led to Aβ precursor protein and Tau protein cleavage, and the Aβ and Tau alterations finally caused cognitive impairment (Wang Z. H. et al., 2019). Many studies reported BDNF levels decreased in AD patients and MCI (Tanila, 2017).

Circulating and central irisin acted on the brain to exert beneficial effects. Irisin bound and modified the function of neurotransmitter receptors in the forebrain, then neurons. The receptor of irisin in the brain was integrin-αV/β5 heterodimers (Jackson et al., 2021). Recombinant irisin stimulated the cAMP/PKA/CREB pathway in human cortical slices (Lourenco et al., 2019). CREB protein is a cellular transcription factor that plays a widely confirmed role in neuronal plasticity and long-term memory formation in the brain (Sen and Stress, 2019). Irisin increased cAMP and phosphorylated CREB (pCREB) in mouse hippocampal slices, which bolstered the expression of BDNF. According to the study by Lourenco et al. (2019), irisin-induced CREB phosphorylation was mediated by PKA. Fahimi et al. (2017) reported that mice after exercises appeared appreciable increase in BDNF mRNA and protein levels, distinctively elevated synaptic load in the dentate gyrus, and increased irisin and TrkB receptor levels in the astrocytes, indicating that irisin might mediate the effects of exercise on brain function and could be a messenger of periphery and central cross talk. Zsuga et al. (2016) proposed that irisin may be a mediator between exercise and reward-related learning and motivation through the irisin-BDNF/TrKB-MEK/ERK-mTOR pathway. The TrKB linked with dopamine 3 (D3) receptor signaling such as PI3/Akt/mTOR pathway was also involved. The two pathways were under the control of BDNF and caused increased dopamine content, neuronal plasticity, and raised neuronal survival (Collo et al., 2014). Moon et al. (2013) described that irisin performed favorable effects on hippocampal neuron proliferation primarily *via* the STAT3 signaling pathway. Activation of STAT3 has been confirmed to correlate with stimulating hippocampal neurogenesis (Jung et al., 2006).

FNDC5 was highly expressed in the brain especially in the hippocampus (Wrann et al., 2013; Lourenco et al., 2019). Neuronal FNDC5 gene expression was also regulated by PGC-1α. The orphan nuclear receptor estrogen-related receptor alpha (ERRα) was a central metabolic regulator interacting with PGC-1α (Schreiber et al., 2004). Wrann et al. (2013) found that ERRα was up-regulated in the hippocampus upon exercise. Furthermore, FNDC5 regulated BDNF gene expression in a cell-autonomous manner, and BDNF decreased FNDC5 gene expression as a part of a potential feedback loop. Elevated expression of FNDC5 strikingly up-regulated BDNF gene expression. Moreover, peripheral delivery of FNDC5 also increased BDNF expression in the hippocampus, and ERK1/2 was a critical regulator of FNDC5 expression and function on neuronal differentiation (Hosseini Farahabadi et al., 2015; Wrann, 2015). In addition to the direct regulation of FNDC5 to BDNF, irisin was also processed from FNDC5 in the hippocampus. Thus, FNDC5/irisin acted as a messenger of muscle brain cross talk, influencing the neurogenesis in cognitive impairment, in particular through the neuroprotective effects of BDNF.

### FNDC5/Irisin and Inflammation

Emerging evidence suggested the importance of inflammation in the pathogenesis of AD and mild cognitive impairment (Holmes, 2013; Shen et al., 2019). According to a meta-analysis of 170 studies, patients with AD and MCI were accompanied with elevated inflammatory markers in both CSF and periphery, such as C-reactive protein (CRP), interleukin-6 (IL-6), soluble tumor necrosis factor receptor 1 (sTNFR1), soluble tumor necrosis factor receptor 2(sTNFR2), alpha1-antichymotrypsin (α1-ACT), IL-1β, soluble CD40 ligand, IL-10, monocyte chemoattractant protein-1 (MCP-1), transforming growth factor-beta 1(TGF-β1), soluble triggering receptor expressed on myeloid cells 2 (sTREM2), and so on (Shen et al., 2019).

The most common neuroinflammation is postoperative. Disruption of the BBB is the hallmark of neuroinflammation; BBB dysfunction like increased BBB permeability has been regarded as accounting for cognitive impairment (Yang et al., 2017). Surgical trauma induced the innate immune system of the brain through the nuclear factor-κB (NF-κB) pathway, leading to endothelial dysfunction and increased permeability of the BBB (Alam et al., 2018). The neuroinflammation consequences included neuronal apoptosis, damaged hippocampal neurogenesis, and impaired synaptic plasticity connections, resulting in neurodegenerative diseases (Zhang et al., 2016; Feng et al., 2017; Alam et al., 2018).

Another type of neuroinflammation is obesity-related inflammation. Obesity is related to chronic low-grade systemic inflammation (Gregor and Hotamisligil, 2011; Spencer, 2013). Inflammatory cascade was initiated by the stimulation of free fatty acid and lipopolysaccharide (LPS) receptor, toll-like receptor 4 (TLR4) on immune cells (Shu et al., 2012). The downstream factors of the TLR family signaling involve the adapter molecule MyD88, which activated NF-κB and MAPK pathways. Both of them were important for the production of cytokines and chemokines (Trinchieri and Sher, 2007; Lim and Staudt, 2013). Maric et al. (2014) suggested that the hypothalamic mRNA expression of IL-1β, IL-6, and TNF-α significantly increased in high-saturated fat (HSF)-diet rats. Qin et al. (2007) investigated that LPS-induced MAPK and STAT-3 activation, as well as the expression of IL-10, made a difference to the suppressor of cytokine signaling 3 (SOCS3) transcription and expression in macrophages and microglia, which alleviated adaptive and innate immune responses. SOCS3 activated the ERK-MAPK pathway, inhibited the NF-κB pathway, and offended cAMP-mediated signaling (Qin et al., 2007). In addition, neuroinflammation was related to microglia 1 (M1), a pro-inflammatory cell, and the anti-inflammatory microglia 2 (M2) (Sica and Mantovani, 2012). Similarly, astrocytes also have two phenotypes, pro-inflammatory astrocytes 1 (A1) and anti-inflammatory astrocytes 2 (A2) (Kwon and Koh, 2020). As a result, the neuroinflammation is under control of the polarization status of M1/M2 and A1/A2.

Irisin has already been confirmed to have anti-inflammatory effects (Pukajło et al., 2015). FNDC5 has been confirmed to attenuate adipose tissue inflammation through the AMPK pathway to induce macrophage polarization in obese mice (Xiong et al., 2018). Irisin prevented LPS-mediated liver injury by inhibiting apoptosis, nod-like receptor pyrin-3 (NLRP3) inflammasome activation, and NF-κB signaling (Li et al., 2021). Mazur-Bialy (2017) demonstrated that irisin not only promoted the activity and proliferation of macrophages and phagocytosis but also attenuated the respiratory burst of macrophages, which increased immunocompetent activity. Mazur-Bialy et al. (2017a) reported that irisin exerted its anti-inflammatory effects by downregulating the NF-κB pathway, reducing TNF-α, IL-6, and MCP-1 in adipocyte 3T3 L1 cell line, thus attenuating the obesity-related neuroinflammation. Irisin was proved to improve memory and cognition in diabetic mice by reducing the expression of IL-1β and IL-6 in the murine hippocampus (Wang K. et al., 2019). The underlying mechanism was by downregulating the P38, STAT3, and NFκB pathways, which was related to the cytokine cascade. The reactive oxygen species-NLRP3 (ROS-NLRP3) pathway was also involved in the inhibition of irisin on the neuroinflammation (Peng J. et al., 2017). Furthermore, irisin played a pivotal role in the phenotypic switch of adipose tissue macrophages from M1 to M2 to regulate neuroinflammation (Dong et al., 2016). Irisin was also involved in autophagy, which affected Tau proteins in dementia (Pesce et al., 2020). Different pathways involved in how irisin affected autophagy, such as the AMPK/SIRT1/PGC-1α pathway in pancreatic β cells in insulin resistance stage (Li Q. et al., 2019), and MAPK pathways in the hepatic I/R injury model (Bi et al., 2020). Although the mechanisms on how irisin directly influenced central autophagy were scarce, there was a consensus on the link between irisin and AMPK. The indirect effects of irisin in autophagy are reliable, and the direct pathway still needs to explore. Table 1 summarizes the experimental studies suggesting the roles of FNDC5/irisin in inflammation.

**TABLE 1:** Experimental studies suggesting the roles of FNDC5/irisin in inflammation.

References	Models	Findings	Pathways
Xiong et al., 2018	HFD-induced obese mice	FNDC5 knock-down ↑inflammation and M2 to M1	Decreasing NF-κB-p65, p38, ERK, and JNK pathways
		FNDC5 overexpression ↓inflammation and ↑M1 to M2	AMPK pathway
	RAW264.7 macrophages	FNDC5↓M1 polarization	NF-κB pathway
Li et al., 2021	LPS-induced liver injury rat; LPS-challenged RAW264.7 cells	Irisin ↓inflammation and apoptosis	NLRP3 inflammasome activation and NF-κB signaling
Mazur-Bialy, 2017	RAW264.7 macrophages	Irisin ↑macrophage activity, proliferation; and phagocytosis ↓macrophage respiratory burst	Reducing ROS overproduction
Mazur-Bialy et al., 2017a	Adipocyte 3T3 L1 cell	Irisin ↓proinflammatory cytokines (TNF-α, IL-6)	NF-κB pathway
		Irisin ↑adiponectin synthesis	
Wang K. et al., 2019	Streptozotocin-induced diabetic mice	Irisin ↑memory and cognitive deficiency; ↓synaptic protein loss; ↓IL-1β and IL-6 levels in Hippocampus and CSF	Reducing the activation of P38, STAT3, and NF-κB pathways
Peng J. et al., 2017	OGD-induced PC12 cell line	Irisin ↓oxidative stress, inflammation, and apoptosis; ↓IL-1β and IL-18; ↓ROS and MDA	NLRP3 inflammatory signaling
Dong et al., 2016	HFD-fed mice	Irisin ↓inflammation; ↑M1 to M2	AMPK and Akt pathway
Li Q. et al., 2019	INS-1 cells	Irisin ↓autophagy; ↑INS-1 cell function and survival	AMPK/SIRT1/PGC-1α pathway
Bi et al., 2020	Hepatic IR old rats	Irisin ↓inflammation	MAPK pathways

*Abbreviations: HFD, high fat diet; LPS, lipopolysaccharide; ROS, reactive oxygen species; NLRP3, NOD-like receptor pyrin 3; OGD, oxygen-glucose deprivation; MDA, malondialdehyde; HepG2, human hepatocellular carcinoma cells; IR, ischemia-reperfusion.*

### FNDC5/Irisin and Oxidative Stress

Oxidative stress is critical in elderly cognitive impairment and AD (Chen and Zhong, 2014). The mechanisms of oxidative stress in AD included mitochondrial dysfunction, metal accumulation, hyperphosphorylated Tau protein, and inflammation. Mitochondrial dysfunction was mainly associated with ROS production resulting from Aβ (Perez Ortiz and Swerdlow, 2019). Increased Aβ1–40 and Aβ1–42 and decreased ATP synthesis and ATPase activity were reported to promote ROS generation in mitochondria (Sharma et al., 2021). Metal ions, such as Cu, Zn, and Fe, were perceived to play a pivotal role in AD (Faller and Hureau, 2012). Metal ions accumulation was also associated with Aβ for its metal binding sites for Zn2+, Cu2+, and Fe3+. Theoretically, Aβ binds to Cu2+ or Fe3+ resulting in reduced Cu+ and Fe2+, respectively. The binding was accompanied by the production of hydrogen peroxide (H2O2), which reacted with Fe2+ to generate Fe3+ and hydroxyl radicals (OH) (Gaeta and Hider, 2005; Chen and Zhong, 2014). Metal mal-metabolism increased the oxidative stress. Violet et al. (2014) suggested the Tau protein alterations contributed to the impaired safeguarding function of DNA and RNA, promoting the aggregation of nucleic acid oxidative damage in the AD brain. Finally, as mentioned before, the inflammation arose the generation of ROS.

FNDC5/irisin has been confirmed the anti-oxidative effects in many studies. Zhang et al. (2020) suggested that FNDC5 decreased ROS production, MDA level, and NADPH oxidase activity *via* its subunit p67phox and increased SOD1 and SOD2 expression in doxorubicin-treated hearts. Besides, FNDC5/irisin exerted the anti-oxidative effects *via* the AKT/GSK3β/FYN/Nrf2 signaling in an mTOR-independent manner. Wang et al. (2020) reported that irisin attenuated oxidative stress *via* 8-OHdG and reversed Sirt3 and UCP-1 pathways to promote mitochondrial membrane potential (MMP), ATP production, and the catalase to alleviate reactive oxygen radical generation, mitochondrial fusion and fission in the osteoarthritis model. Irisin targeted mitochondria to promote SOD-2 activity and prevented the loss of MMP, decreased the ROS activity, and finally relieved the oxidative stress in the ischemia/reperfusion (I/R) heart (Wang et al., 2018). Besides, in an ischemia/reperfusion (I/R) liver model, irisin was shown to reduce oxidative stress *via* improving UCP-2 expression, which led to reduced ROS production, restrained mitochondrial fission, and increased mitochondrial DNA copy to improve mitochondrial biogenesis (Bi et al., 2019). The Nrf2/HO-1/HMGB1 signaling participated in the anti-oxidative performance of irisin, increasing the expression of anti-oxidative factors such as SOD-1, glutathione peroxidase (GPx), and catalase-9 (Cat-9) (Mazur-Bialy and Pocheć, 2021). Activation of the AMPK-Sirt1-PGC-1α pathway and Akt/ERK1/2 pathway were involved in the irisin’s anti-oxidative effect (Li et al., 2017; Wu et al., 2020). Table 2 summarizes the experimental studies suggesting the roles of FNDC5/irisin in oxidative stress.

**TABLE 2:** Experimental studies suggesting the roles of FNDC5/irisin in oxidative stress.

References	Models	Findings	Pathways
Zhang et al., 2020	DOX-induced Mice; DOX-induced H9C2 cells	FNDC5 ↓cardiac oxidative damage	AKT/GSK3β/FYN/Nrf2 signaling
		FNDC5 ↓cardiomyocyte apoptosis	AKT/mTOR signaling
Wang et al., 2020	DMM-induced OA mice	Irisin ↓autophagy and apoptosis;	PGC-1α; UCP-1; Sirt3
Wang et al., 2018	Myocardial I/R mice; A/R injury H9c2 cells	Irisin ↓apoptosis; ↓MMP loss; protects against I/R-injured myocardium	SOD2 targeting to mitochondria
Bi et al., 2019	Hepatic I/R Mice; H/R injury HL-7702 cell	Serum irisin increased after ischemia and 4 h after reperfusion then decreased.	PGC-1α; UCP 2; Fis-1;Drp-1
		Irisin ↓organ injury and apoptosis; ↓inflammation; ↓excessive mitochondrial fission; ↑mitochondrial biogenesis; ↓oxidative stress (↓liver MDA level)	
Mazur-Bialy and Pocheć, 2021	LPS-induced RAW264.7 macrophages	Irisin ↓respiratory burst and apoptosis; ↑Nrf2, HO-1 SOD1, SOD2, GPx, Cat-9; ↓HMGB1	Nrf2/HO-1/HMGB1 pathway
Wu et al., 2020	alcat1 knockout MI Mice; NRK cells treated with H2O2	Irisin ↓oxidative stress and apoptosis in NRK cells	AMPK-Sirt1-PGC-1α pathway
Li et al., 2017	MCAO Mice; PC12 neuronal cells with OGD	Plasma irisin levels are negatively associated with brain infarct volume, neurological deficit and inflammation.	Akt and ERK1/2 pathways
		Irisin ↓ inflammation and oxidative stress	

*Abbreviations: DOX, doxorubicin; DMM, destabilized medial meniscus; OA, osteoarthritis; I/R, ischemia/reperfusion; A/R, anoxia/reoxygenation; SOD, superoxide dismutase; H/R, hypoxia/reoxygenation; UCP, uncoupling proteins; Drp-1, dynamin related protein 1; Fis-1, fission 1; GPx, glutathione peroxidase; Cat-9, catalase-9; HMGB1, high-mobility group box 1; Nrf2, nuclear factor erythroid 2-related factor 2; HO-1, heme oxygenase-1; NRK, normal rat kidney; ALCAT1, acyltransferase1; MCAO, middle cerebral artery occlusion; OGD, oxygen and glucose deprivation.*

## FNDC5/Irisin Act on Dementia-Related Disease

### FNDC5/Irisin and Coronary Artery Disease

Coronary artery disease (CAD) was associated with dementia as they shared common risk factors such as aging, obesity, type 2 diabetes (T2DM), and hypercholesterolemia. The prevalence of both dementia and CAD increases with age, with the prevalence of dementia in those with acute myocardial infarction (AMI) increasing from 1.2% in those aged 65–69 years to 14.8% in those aged above 85 years (Fowkes et al., 2016).

Various studies suggested serum irisin levels were decreased in patients with CAD, indicating the positive effects of irisin on CAD (Khorasani et al., 2019; Wang S. et al., 2019; Guo et al., 2020). In a myocardial infarction (MI) mouse model, irisin appeared to suppress cardiomyocyte apoptosis and fibrosis and promote angiogenesis *via* the ERK signaling, which collectively improved the cardiac function and reduced the infarct size of the post-MI model (Liao et al., 2019). Zhao et al. (2016) found that in histone deacetylases (HDAC)-over-expressed H9c2 cardio-myoblasts that went through hypoxia/reoxygenation-induced injury, irisin treatment increased cardio-myoblast survival and decreased the LDH release to alleviate cytotoxicity. Besides, irisin repressed the cell apoptosis *via* reducing active-caspase 3 and annexin V signals, mitigating the loss of MMP to protect mitochondrial damage. Furthermore, irisin held back the opening of mitochondrial permeability transition pore, which was critical for myocardial injury.

### FNDC5/Irisin and Hypertension

Hypertension is associated with an increased incidence of vascular dementia (Sharp et al., 2011). Midlife systolic blood pressure (SBP) was suggested to be a significant predictor of cognition that deficits later in life (Launer et al., 1995). In the elderly, dysfunction of cerebral autoregulation led to vulnerable cerebral hemodynamics. Autoregulation protected the brain from hypertension but increased the risk of cerebral hypotension. Inappropriate antihypertensive therapy might further increase the risk of chronic cerebral hypoperfusion and subsequent dementia (Feldstein, 2012). Higher diastolic blood pressure (DBP) and lower SBP were correlated with impaired cognition (Nilsson et al., 2007; Tsivgoulis et al., 2009).

Irisin made a difference in regulating blood pressure through central and peripheral pathways; central irisin increased cardiac output and blood pressure by activating hypothalamic paraventricular nucleus of the hypothalamus (PVN) neurons, while peripheral irisin secreted from skeletal muscle reduced blood pressure *via* Adenosine triphosphate-sensitive potassium (KATP) channels to dilate vessels (Zhang et al., 2015). Besides, Irisin improved hypertension by protecting endothelial function *via* the AMPK-Akt-eNOS-NO and Nrf2 signaling pathway, the Nrf2 signaling pathway also participated in alleviating oxidative stress in the hypothalamus (Fu et al., 2016; Huo et al., 2020). Huang et al. (2022) proposed that irisin inhibited the NF-κB signaling pathway to lower blood pressure, along with reduced angiotensin II type 1 receptor (AT1R) expression and function.

### FNDC5/Irisin and Heart Failure

A considerable number of patients with heart failure (HF) have cognitive problems (Cannon et al., 2017). Vascular dysfunction and loss of cardiac perfusion pump function can trigger the typical AD feature such as Aβ accumulation and hyperphosphorylated Tau tangles, as HF and AD shares common risk factors like inflammation and oxidative stress (Daniele et al., 2020).

Irisin exerted positive influences on mitochondrial dysfunction, oxidative stress, metabolic imbalance, and energy expenditure in HF (Ho and Wang, 2021). Cohort and experimental studies were conducted to elucidate the correlation between irisin and HF. Several cohorts showed increased serum irisin levels in patients with HF (Shen et al., 2017; Kalkan et al., 2018; Abd El-Mottaleb et al., 2019). Peng Q. et al. (2017) suggested that irisin ameliorated H2O2-induced apoptosis in H9c2 cardio-myoblasts and improved cell viability *via* miR-19b/PTEN/AKT/mTOR pathway. Li R. et al. (2019) found that irisin-induced protective autophagy and alleviated apoptosis signaling attenuated the myocardial hypertrophy and cardiomyocytes apoptosis. The AMPK-ULK1 pathway might be involved in the underlying mechanisms (Li et al., 2018).

### FNDC5/Irisin and Stroke

Stroke is a pronounced disease related to cognition impairment and contributes to damaged life quality (Obaid et al., 2020). Stroke is divided into the ischemic and hemorrhagic stroke, the former makes up 85% (Amarenco et al., 2009; Beal, 2010). A total of 23.9% of older stroke survivors developed dementia (Allan et al., 2011). Taking ischemic stroke as an example, brain injury secondary to the stroke was a result of the post-stroke excitotoxicity, oxidative and nitrative stress, inflammation, and apoptosis (Khoshnam et al., 2017). Besides, Goulay et al. (2020) have reported that stroke exacerbated the deposition of Aβ.

Irisin has been reported to perform neuroprotective effects on stroke (Liu et al., 2020). Irisin mitigated brain injury after stroke *via* inhibiting inflammation and oxidative stress and preventing BBB dysfunction (Peng J. et al., 2017; Guo et al., 2019). Jin et al. (2019) suggested that irisin attenuated the brain injury after the cerebral ischemia/reperfusion (I/R) injury especially in the hippocampus region through the Notch signaling pathway. Irisin promoted the Notch1, Notch intracellular domain (NICD), and Hes1 expression, which were reported to exert effects in AD and other neurodegenerative diseases. Irisin alleviated neuronal apoptosis, accompanied by decreasing the caspase-3 expression, a critical apoptotic effector. Besides, irisin reduced the inflammation, decreasing the TNF-α and IL-1β levels (Berezovska et al., 1998; Alberi et al., 2013). Yu et al. (2020) reported that irisin protected the neurological function in a middle cerebral artery occlusion (MCAO) I/R injury model *via* suppressing the TLR4 and NF-κB pathways. Others also elucidated the neuroprotective effects of irisin in mice with MCAO and OGD neuronal cells *via* Akt and ERK1/2 signaling pathways (Li et al., 2017). Irisin relieved the post-ischemic inflammation by downregulating TNF-α and IL-6 expression, suppressed the microglial infiltration, and decreased the MPO-1+ cell numbers, as well as reduced the post-ischemic oxidative stress by decreasing the levels of 4-HNE and MDA. Furthermore, mitochondrial dynamics were involved in the ischemic stroke, and mitochondrial defects are critical for AD (Yan et al., 2013; Anzell et al., 2018). Irisin improved mitochondrial function *via* AMPK pathway as the AMPK was a guardian of mitochondrial homeostasis (Tang et al., 2016; Herzig and Shaw, 2018; Siteneski et al., 2018; Xin et al., 2020). In summary, irisin exerted neuroprotective effects after stroke to prevent cognitive impairment primarily through its anti-inflammatory and anti-oxidative effects, as well as the beneficial effects on mitochondria.

### FNDC5/Irisin and Parkinson’s Disease

Parkinson’s disease is the second most frequent senile neurodegenerative disease (Mhyre et al., 2012). Patients with PD often developed cognitive deficits and dementia, especially in elderly patients (Aarsland et al., 2017). PD-dementia is a classic type of dementia.

Irisin played a protective role in PD. In a mouse model of PD, irisin treatment prevented dopaminergic neurons from apoptosis and degeneration (Zarbakhsh et al., 2019). Mahalakshmi et al. (2020) elucidated the benefits of exercise on PD, and irisin was a mediator of exercise-induced BDNF. Raefsky and Mattson (2017) suggested that irisin might protect neuronal mitochondria function in PD *via* antioxidation, autophagy, and DNA repair regulations.

### FNDC5/Irisin and Depression

Depression and dementia often occur at the same time in the elderly (Bennett and Thomas, 2014). Depression is both the risk factor and prodrome of dementia (Gutzmann and Qazi, 2015). The interreaction of depression and dementia is complex.

Irisin improved depressive neuropathology by regulating mitochondria function *via* PGC-1α signaling and modifying synaptic plasticity *via* BDNF signaling (Jo and Song, 2021). Hou et al. (2020) proposed that irisin attenuated the postoperative depressive-like behavior and reduced neuron death and cytokines release from astrocytes through inhibiting the surface expression of epidermal growth factor receptors (EGFR) in the mice model. Siteneski et al. (2018) also suggested that central irisin administration manifested antidepression effect, associated with the adjustment of gene expression of PGC-1α, FNDC5, and BDNF in the hippocampus and prefrontal cortex of mice.

## Future Direction

There is a long way to intervene and delay the progression of elderly cognitive impairment. Based on the irisin secretion and function to optimize the exercise protocol such as the amount of exercise, the form of exercise, and the duration of exercise, further research is needed. Factors affecting exercise, such as age, frailty, sarcopenia, and fracture, also need to be considered. Although many studies have been reported to support the favorable effects of FNDC5/irisin, there are some limitations. Many studies are based on experimental studies, and direct studies of irisin on central autophagy are scarce. Besides, the difference of plasma irisin levels alterations in patients with dementia was not significant and has not reached a consensus. Interfering factors such as age, gender, race, and disease duration differences cannot be ignored. There are also some controversial results and views. On the one hand, Raschke et al. (2013) argued that the beneficial effect of irisin observed in mice can be translated to humans. Although there are many registered clinical trials to clarify the effects of irisin on the human body, large-scale clinical research and long-term follow-up are required to study the relationship between FNDC5/irisin and cognition. Besides, to carry out the animal experiments and clinical research simultaneously and to conduct comparative analysis are very necessary to elucidate the difference in FNDC5/irisin effects in the mice and human body. On the other hand, the current irisin detection method is still insufficient. ELISA has been widely used in the examination of irisin levels in serum or other specimens in humans and animals. However, Albrecht et al. (2015) argued that ELISA kit for irisin may not be accurate. Besides, ELISA can be influenced by a series of factors such as preservation conditions, temperature, antibody, and operational contingency. As a result of the conflicting opinions, conducting comparative studies on the sensitivity and specificity of current ELISA kits is a research direction. A high-quality meta-analysis or systematic review of the efficacy of ELISA kits for irisin also can be considered. Most of the ELISA kits for irisin were for laboratory research only, not for drug, diagnostic, or other use. Exploring new methods with high sensitivity and specificity as well as diagnostic value in clinical conditions is also the direction of future efforts, such as the application of sensors or nanotechnology.

## Conclusion

Cognitive impairment is a worldwide public health problem, which seriously affects the quality of life of the elderly and increases the burden of care. Clarifying the pathological mechanism of dementia and exploring drugs to prevent, treat, and delay the course of dementia have always been the direction of efforts. Physical exercise and lifestyle are believed to defend against cognitive decline in the elderly. Irisin might be a mediator of muscle and brain cross talk mainly through the PGC-1α/FNDC5/BDNF pathway. More information is needed to optimize exercise protocols based on irisin for patients with dementia. Our review discussed the favorable effects of irisin on cognitive impairment, such as the positive effect irisin on neurogenesis and synapse; anti-inflammatory and anti-oxidative effects; and possible connections of irisin on dementia-related diseases such as CAD, hypertension, HF, stroke, PD, and depression. The serum irisin level alterations in dementia have not reached a consensus. Large-scale clinical research and long-term follow-up are required to explore whether serum irisin is a diagnostic or prognostic factor for dementia. The current detection method for irisin is still limited to ELISA. It is also an exploratory direction to find more sensitive, specific, and simple detection methods.

## Author Contributions

JP participated in literature collection, preparation, and wrote the draft. JW supervised the whole project. Both authors participated in the conception and study design, contributed to the manuscript revision, and approved the submitted version.

## Conflict of Interest

The authors declare that the research was conducted in the absence of any commercial or financial relationships that could be construed as a potential conflict of interest.

## Publisher’s Note

All claims expressed in this article are solely those of the authors and do not necessarily represent those of their affiliated organizations, or those of the publisher, the editors and the reviewers. Any product that may be evaluated in this article, or claim that may be made by its manufacturer, is not guaranteed or endorsed by the publisher.
